# Simultaneous RPHPLC Determination of Nitazoxanide and Ofloxacin in Combined Tablet Dosage Form

**DOI:** 10.4103/0250-474X.44600

**Published:** 2008

**Authors:** R. R. Kalta, R. Sharma, S. C. Chaturvedi

**Affiliations:** School of Pharmacy, Devi Ahilya Vishwa Vidyalaya, Takshashila Campus, Khandwa Road (Ring Road), Indore-452 017, India

**Keywords:** Nitazoxanide, ofloxacin, RP-HPLC, simultaneous estimation

## Abstract

A simple, precise, accurate, rapid and reproducible reverse phase high performance liquid chromatographic procedure was developed for simultaneous determination of nitazoxanide and ofloxacin in tablet dosage form at a single wavelength. The mobile phase used was a combination of acetonitrile:0.25M potassium dihydrogen phosphate buffer (80:20) with 0.5%v/v of triethylamine and the pH was adjusted to 2.5 by adding orthophosphoric acid. The detection of the combined dosage form was carried out at 320 nm and flow rate was set to 1ml/min. Linearity was obtained in the concentration range of 5 to 25 μg/ml of nitazoxanide and ofloxacin with correlation coefficients of 0.9987 and 0.9995, respectively. The results of the analysis were validated statistically and recovery studies confirmed the accuracy of the proposed method.

Nitazoxanide (NTZ), chemically N-(5-nitro-2-thiazoly) salicylamide acetate^1^, is an antiamoebic and anthelmintic agent. It is indicated for amoebiasis, helminthiasis, giardiasis, fascioliasis, trichomoniasis and cryptosporidiosis, including those with AIDS or HIV infections^2^,^3^. It is not yet official in any of the pharmacopoeia. Literature survey revealed RP-HPLC method for its determination in bulk drug and pharmaceutical formulation^4^. Ofloxacin (OFX), 9-fluoro-2,3-dihydro-3-methyl-10-(4-methyl-1-piperazinyl)-7-oxo-7H-pyrido(1,2,3-di)-1,4-benzoxazine-6-carboxylic acid^5^, is useful in the treatment of genitourinary, respiratory, gastrointestinal, skin and soft tissue infections, peritonitis and gonorhoea^6^. Few UV spectrophotometric methods and HPLC methods have been reported for the estimation of ofloxacin, especially in body fluids and blood plasma as well as from pharmaceutical preparations 7–11. OFX is official in BP and USP NF^12^,^13^. Literature review revealed that no HPLC method is yet reported for the simultaneous estimation of the NTZ and OFX in combined tablet dosage form at single wavelength. Therefore, it was thought worthwhile to develop simple, precise, accurate reverse phase HPLC method for simultaneous estimation of NTZ and OFX in combined tablet dosage form.

Pharmaceutical grade NTZ (Batch No. SNT 0606023) and OFX (Batch No. KYOFM 20050852) were supplied as a gift sample by Ind-Swift Laboratories Limited, Samba, (Jammu and Kashmir), India. The tablet dosage form (Nitazet-O, Batch No. 6GCT 37, Mfg. Dt. 07/2006 and Exp. Dt. 06/2008) was procured from the local market (Label claim: 500 mg NTZ and 200 mg OFX) marketed by Glenmark Pharmaceuticals Limited, Mumbai. All chemicals used were of HPLC grade and were purchased from Spectrochem, Mumbai, India.

LC system (Shimadzu LC 10AT VP HPLC) used consist of pump with universal loop injector (Rheodyne 7725) of injection capacity 20 μl. Detector consists of photodiode array detector SPD-10 AVP, Shimadzu; the reversed phase column used was Luna C_18_ (5 μm, 25 cm × 4.6 mm i.d.) Phenomenex, USA, at ambient temperature. Among the several mobile phases used for the simultaneous estimation of NTZ and OFX, acetonitrile:0.25 M potassium dihydrogen phosphate buffer (80:20) with 0.5%v/v of triethylamine, adjusted to pH 2.5 using orthophosphoric acid was found to be most suitable and was filtered through 0.2 micron membrane filter.

Stock solutions of NTZ and OFX 1mg/ml concentration were prepared in mobile phase. 50 mg of NTZ was dissolved in 10 ml of acetonitrile and diluted to 50 ml by the mobile phase, and concentration of 1 mg/ml was obtained. They were further diluted with mobile phase to obtained required concentration of 5, 10, 15, 20, 25 μg/ml. In the similar way 50 mg of OFX was dissolved in 10 ml of buffer and further diluted with 40 ml of mobile phase, and the concentration of 1 mg/ml was obtained. They were further diluted with mobile phase to obtained required concentration of 5, 10, 15, 20, 25 μg/ml. All solutions were stored at room temperature; these solutions were shown to be stable during the period of study.

A volume of 20 μl of each standard after filtration by 0.2-micron membrane filter was injected into column. All measurements were repeated five times for each concentration and respective calibration curves were constructed by plotting the peak area versus the corresponding drug concentration. The slope and co-rrelation coefficients were determined (Table 1).

**TABLE 1:** RESULTS OF VALIDATION STUDIES

SST and other parameters	NTZ	OFX
*Theoretical Plates (N)	5889	3119
*Resolution (R_s_)	7.17	--
Linearity Range (μg/ml)	5-25	5-25
Percentage Recovery (%)	99-102	99-102
Drug Recovered (500:200 mg)	200.03	500.09
LOD (μg/ml	0.14	0.59
LOQ (μg/ml)	0.43	1.80
*Tailing Factor	1.37	1.40
*Capacity Factor	0.55	--
*Retention Time (Minutes)	3.38	2.19
Slope (m) in Tablet Form	28503	32194
Intercept (b) in Tablet Form	123909	108727
Co-relation coefficient	0.9987	0.9995
Specificity/selectivity	No interference	No interference
Stability of sample solution	>24 hours	>24 hours

SST: System suitability test

* calculated at 5% peak height, LOD: Limit of detection, LOQ: Limit of quantitation

To determine the content of NTZ and OFX in tablet dosage form (Label claim: 500 mg of NTZ and 200 mg of OFX); twenty tablets were weighed; their average weight was determined and finely powdered. Then triturate tablet dosage form equivalent to 50 mg of NTZ was taken and was dissolved in 10 ml of mobile phase by stirring for 2 min. and the volume was made up to 50 ml by mobile phase. Then 1 ml from that solution was taken and diluted with mobile phase to make up to 10 ml. The final solution was filtered by 0.2-micron membrane filter by using injection filter. Then with the help of pipette 0.5, 1.0, 1.5, 2.0, 2.5 ml of the filtered solution taken in small test tubes and diluted up to 10 ml of each respectively which contain 5:2, 10:4, 15:6, 20:8, and 25:10 μg/ml of NTZ and OFX respectively. A 20 μl of the above dilutions were injected one by one to the HPLC with the help of Hamilton Syringe. The results are reported in Table 2 and the amount of both the drugs was determined.

**TABLE 2:** RESULTS OF ANALYSIS OF TABLET FORMULATION

Drug	Conc taken (μg/ml)	Conc found (μg/ml)	SD	% RSD	Found (Label claim)	
					
					%	mg
NTZ	5	5.005	0.028	0.56	101.00	500.05
(Label claim: 500mg)	10	10.002	0.018	0.18	100.02	500.10
	15	15.007	0.012	0.08	100.04	500.25
	20	20.000	0.014	0.07	100.00	500.02
	25	25.002	0.142	0.57	100.08	500.04
OFX	2	2.000	0.004	0.23	100.00	200.03
(Label claim: 200mg)	4	4.001	0.002	0.07	100.02	200.05
	6	6.000	0.063	1.05	100.00	200.03
	8	8.000	0.116	1.45	100.00	200.01
	10	10.002	0.112	1.15	100.02	200.04

Conc: concentration, SD: standard deviation, RSD: relative standard deviation.*results are mean of five replications.

To check the accuracy of the developed methods and to study the interference of formulation additives, analytical recovery experiments were carried out by standard addition method. From the total amount of drug found, the percentage recovery was calculated. The results of the analysis are reported in Table 3 and 4.

**TABLE 3:** RESULTS OF RECOVERY STUDY OF NTZ AND OFX

Drug	Initial Conc. (μg/ml)	Conc. added (μg/ml)			% Recover		
			
		A	B	C	A	B	C
NTZ	5	2	4	5	99.06	99.09	101.40
	10	2	4	5	100.58	100.42	100.86
	15	2	4	5	100.41	100.94	100.42
	20	2	4	5	99.68	99.45	99.44
	25	2	4	5	99.62	100.31	101.51
OFX	2	5	10	15	101.28	100.75	100.47
	4	5	10	15	101.40	100.42	99.94
	6	5	10	15	100.81	100.12	99.61
	8	5	10	15	99.84	99.05	100.43
	10	5	10	15	99.60	99.60	101.60

Conc.: Concentration, results are mean of five replicates; Percentage recovery is more than 99%, hence method is accurate and precise.

**TABLE 4:** REPEATABILITY, ACCURACY, PRECISION STUDIES OF NTZ AND OFX

Std.	NTZ			OFX		
		
Conc	Mean peak area*	SD	%RSD	Mean peak area*	SD	%RSD
5	247385.00	636.72	0.25	273000.00	2957.70	1.08
10	406000.00	4606.09	1.13	408890.00	1044.12	0.25
15	549718.00	1058.23	0.19	574000.00	7978.60	1.39
20	689754.00	683.37	0.09	729000.30	2957.70	0.45
25	829289.00	2957.70	0.35	934558.00	2457.02	0.26

Std, Conc: Standard concentration, SD: standard deviation, RSD: relative standard deviation

* Mean of three peak areas.

Optimization of mobile phase and flow rate were performed based on peak parameters such as height, tailing factor, theoretical plates, capacity factor, run time and resolution. The mobile phase acetonitrile:0.25 M potassium dihydrogen phosphate buffer (80:20) with 0.5%v/v of triethylamine, adjusted to pH 2.5 using orthophosphoric acid was found to be satisfactory and gave two symmetrical and well resolved peaks for NTZ and OFX. The resolution between NTZ and OFX was found to be 7.17, which indicates good separation for both the compounds. The retention time for NTZ and OFX were 3.38 min and 2.19 min, respectively (fig. 1). The wavelength 320 nm was selected for simultaneous determination based on maximum absorption of both drugs and best detector response at this wavelength.

**Fig. 1:**
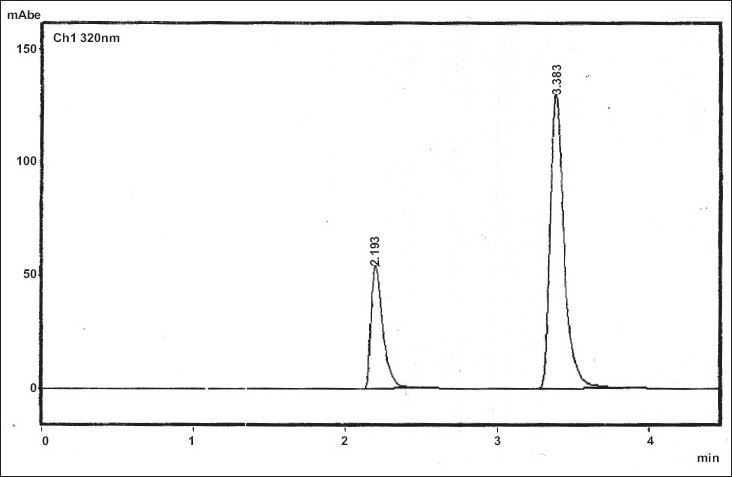
Typical Chromatogram of NTZ and OFX. Chromatogram showing retention time, 2.193 and 3.383 for nitazoxanide (NTZ) and oflaxacin (OFX) in tablet dosage form respectively.

The calibration curve for NTZ and OFX was obtained by plotting the peak area of NTZ and OFX versus their concentration over the range of 5-25 μg/ml and were found to be linear with r=0.9987 and 0.9995 for NTZ and OFX, respectively. The detection limit for NTZ and OFX were 0.14 μg/ml and 0.59 μg/ml, respectively. The quantitation limit for NTZ and OFX were 0.43 μg/ml and 1.80 μg/ml, respectively, which suggested that a nanogram quantity of both the compounds can be estimated accurately. The validation and system suitability parameters are shown in Table 1. The recoveries of NTZ and OFX were found to be in the range 99.06-100.58% and 99.05-101.60%, respectively (Table 3). Repeatability, accuracy and precision data are reported in Table 4.

The liquid chromatographic method was applied for the simultaneous determination of NTZ and OFX in combined tablet dosage form. The result of analysis of tablet formulation containing 5 to 25 mg of NTZ and 2 to 10 mg of OFX revel SD ranging from 0.012 to 0.142 and%RSD ranging from 0.07 to 0.57 in the case of NTZ and 0.002 to 0.116 and 0.23 to 1.15 in the case of OFX, all values being mean of five replicates (Table 2).

Proposed method describes a new RPHPLC method for the determination of NTZ and OFX in combined tablet dosage form. The method gives good resolution between both the compounds and with a short analysis time (<5 min). The method was validated and found to be simple, sensitive, accurate and precise. Percentage of recovery shows that the method is free from interference of the excipient used in the formulation. Therefore, the proposed method can be used for routine analysis of NTZ and OFX in their combined tablet dosage form.
